# The Effect of Diamond Bur Wear During Grinding on the Marginal Gap of Zirconia Lithium Silicate Single Crowns: An In Vitro SEM Analysis

**DOI:** 10.3390/dj14050291

**Published:** 2026-05-12

**Authors:** Roi Avrahami, Joseph Nissan, Ophir Rosner, Alexandra Andronik, Diva Lugassy, Gil Ben-Izhack

**Affiliations:** 1 Department of Oral Rehabilitation, The Maurice and Gabriela Goldschleger School of Dental Medicine, Gray Faculty of Medicine & Health Sciences, Tel Aviv University, Tel Aviv 6997801, Israel; roiavrahami@mail.tau.ac.il (R.A.); nissandr@tauex.tau.ac.il (J.N.); ophirr@tauex.ac.il (O.R.); andronik@mail.tau.ac.il (A.A.); 2 Department of Orthodontics, The Maurice and Gabriela Goldschleger School of Dental Medicine, Gray Faculty of Medicine & Health Sciences, Tel Aviv University, Tel Aviv 6997801, Israel; divaluga@mail.tau.ac.il; 3 Dental Division, Shamir (Assaf Harofeh) Medical Center, Zerifin 70300, Israel

**Keywords:** CAD/CAM, marginal gap, bur wear, zirconia-reinforced lithium silicate, SEM, digital dentistry

## Abstract

**Objectives**: In this in vitro study, we aimed to evaluate the effect of bur wear during grinding on the marginal gap of Zirconia Lithium Silicate (ZLS) single crowns. **Methods**: A single maxillary right canine typodont tooth, pre-prepared for a full ceramic crown, was scanned using an intra-oral scanner. A total of 32 ZLS crowns were ground using a four-axis grinding unit and divided into four equal groups (*n* = 8) based on the grinding sequence to represent progressive bur wear: Group 1 (crowns 1–8), Group 2 (crowns 9–16), Group 3 (crowns 17–24), and Group 4 (crowns 25–32). All crowns were ground consecutively using the same set of diamond burs. Each crown was temporarily cemented to the typodont tooth, and the marginal gap was measured at four reference points (buccal, palatal, mesial, distal) using scanning electron microscopy (SEM) at ×250 magnification. A Kolmogorov–Smirnov test performed on the study variables indicated a normal distribution (*p* > 0.05). **Results**: One-way ANOVA revealed no significant differences in the mean total marginal gap between the groups (*p* = 0.117), with values of 47.20 ± 5.16, 44.62 ± 7.23, 54.14 ± 10.02, and 48.53 ± 7.87 μm for Groups 1, 2, 3, and 4, respectively. Furthermore, no significant differences were found regarding a specific surface (distal, mesial, palatal, buccal) (*p* > 0.05). **Conclusions**: The cumulative wear of diamond burs after the consecutive grinding of 32 ZLS crowns did not significantly affect the marginal adaptation. All recorded marginal gaps were well within the clinically acceptable range (<120 μm).

## 1. Introduction

The integration of chairside computer-aided design and computer-aided manufacturing (CAD/CAM) systems has revolutionized restorative dentistry by enabling same-day delivery of high-quality ceramic restorations. These digital systems allow practitioners to design and manufacture indirect restorations in a single clinical visit, significantly reducing traditional laboratory-related delays. However, the precision and clinical success of these chairside-produced restorations depend heavily on the condition and performance of the milling and grinding tools [1,2,3].

The efficiency of this digital workflow begins with high-precision intra-oral scanners (IOSs), such as the Primescan by Dentsply Sirona (Dentsply Sirona, Milford, DE, USA) [4]. This scanner utilizes parallel confocal imaging, a method involving laser and optical scanning to capture multiple cross-sections at various depths, resulting in a detailed three-dimensional (3D) digital model. The captured data is processed into a standard tessellation language (STL) file, which conveys the exact spatial geometry required for restoration design [5]. In the chairside environment, four-axis units like the CEREC MC XL (CEREC^®^ MCXL; Dentsply Sirona, Milford, DE, USA) are widely used due to their clinical adequacy for fabricating single crowns and veneers [6]. These machines typically employ a dual-instrument strategy: a stepped bur for the intaglio surface and a pointed cylinder bur for the external anatomy [7].

The Cerec MC XL is a four-axis wet grinding installation with inLab custom CAD interface software 22.0 in which STL data can be imported. It comes with the proprietary CAM program in Lab CAM but is also provided with an open CAM mode interface. These systems utilize computerized grinding units that grind prefabricated blocks or disks of various materials, such as zirconia, polymethyl methacrylate (PMMA), composites, hybrid ceramics, glass ceramics, lithium disilicate ceramics, and sintered Co-Cr alloys. Digital impressions, including those for individual teeth or fixed partial dentures, have been shown to be highly accurate, highlighting the effectiveness of the scanning phase. The replacement of burs and prosthetic materials is carried out manually [8].

Zirconia-reinforced Lithium Silicate (ZLS), specifically Celtra Duo by Dentsply Sirona (CELTRA^®^ DUO, Dentsply Sirona, Milford, DE, USA) [9], features a microstructure of a homogeneous glassy matrix containing a crystalline component made of round and submicrometric elongated grains of lithium metasilicate and lithium orthophosphate, reinforced with 10% tetragonal zirconia fillers to enhance strength. Lithium disilicate grains are formed following a crystallization process, and there are lithium metasilicate crystallites within the glassy phase in Celtra Duo (about 1 μm). ZLSs are available in a pre-crystallized or crystallized form [10,11].

Previous investigations have extensively evaluated the marginal and internal fit of CAD/CAM-fabricated ceramic restorations, particularly zirconia and ZLS materials [12,13]. Several studies have demonstrated that CAD/CAM-related parameters, including preparation design, cement spacer settings, and milling system characteristics, play a significant role in determining marginal accuracy [14,15]. In addition, growing attention has been directed toward the manufacturing phase, specifically the effect of milling bur degradation. Evidence suggests that progressive diamond bur wear can adversely affect the trueness, precision, and surface quality of milled restorations [16].

A previous study demonstrated that progressive bur wear significantly influences the trueness and precision of ZLS crowns when evaluated using three-dimensional (3D) superimposition techniques [16]. In this context, accuracy is defined by two components: trueness, representing the deviation from the true geometry, and precision, reflecting the reproducibility of repeated measurements [17]. However, while 3D deviation analysis provides a comprehensive assessment of overall accuracy, it does not specifically address marginal adaptation, which remains the most clinically relevant parameter. Marginal discrepancies exceeding 120 μm have been associated with cement dissolution, microleakage, plaque accumulation, and secondary caries development [18].

When examining manufacturer recommendations (Dentsply Sirona) regarding bur replacement, it is mentioned that the type of material has a direct impact on the longevity of the burs, and, specifically for ZLS, a four-axis milling machine should be replaced after approximately 32 units.

Tan and Dudley examined the effect of sequentially milling lithium disilicate (LDS) on the marginal gap when using two different types of milling units (4- and 5-axis); 12 crowns were milled in each group, and the mean marginal gaps were 100.40 μm (4-axis) and 101.08 μm (5-axis), with no significant difference between the two types of milling units. The precision of the crown margins also decreased steadily as the milling burs aged, showing a clear link between tool wear and larger gaps [19].

Despite the increasing body of literature addressing CAD/CAM accuracy and bur wear, there is a notable lack of studies specifically investigating the direct effect of progressive bur wear on the marginal gap of ZLS restorations. Therefore, the aim of this in vitro study was to evaluate this effect on the marginal gap of Zirconia-reinforced Lithium Silicate (ZLS) crowns using scanning electron microscopy (SEM) analysis. The null hypothesis was that progressive bur wear does not significantly affect marginal adaptation.

## 2. Materials and Methods

### 2.1. Study Design

In this in vitro study, Zirconia-reinforced Lithium Silicate (ZLS) (CELTRA^®^ DUO, Dentsply Sirona, Milford, DE, USA) single crowns (n = 32) were ground by a 4-axis machine (CEREC MC XL^®^; Dentsply Sirona). The wear percentage of the burs was documented according to the parameters displayed in the computer software until the system alerted to the failure of the burs or asked to replace them. Each crown was fixed, and the marginal gap was measured by SEM (JSM-IT100; JEOL, Akishima, Tokyo, Japan ) at 4 points of interest (3 measurements at each location and a total of 12 points for each crown) with ×250 magnification, recorded and checked in correlation with the wear percentage of the burs, and compared regarding the mean marginal gap values.

### 2.2. Specimen Preparation

**Figure 1:**
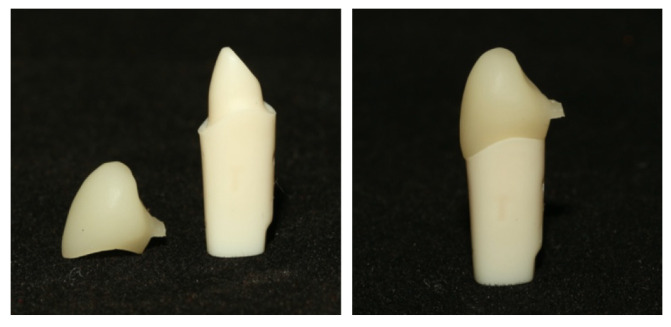
Plastic tooth of the first maxillary canine and plastic tooth with ZLS crown.

A plastic typodont right upper canine tooth (UR31C Jacket; Nissin Dental Products INC., Kyoto, PA, Japan) was prepared as a single abutment for a single full ZLS crown. The tooth was machine-prepared by the manufacturer to ensure standardization for single crown restorations and followed standardized parameters: a 1.2 mm circumferential shoulder finish line, a 6-degree axial convergence angle, and an incisal reduction of 2 mm [20] (Figure 1).

The prepared tooth was scanned using an intra-oral scanner (CEREC^®^ Primescan; Dentsply Sirona, Milford, DE, USA), and the resulting scan was converted into a standard tessellation language (STL) file and processed using CAD software (CEREC SW 5.1.3). The design was made once and was defined as the master design for the full crown. The crown was created with the following virtual parameters: radial spacer, 90 μm; incisal spacer, 90 μm; proximal contact strength, 25 μm; incisal contact strength, 25 μm; dynamic contact strength, 25 μm; radial minimum thickness, 1000 μm; incisal minimum thickness, 1500 μm; margin thickness, 50 μm; margin ramp width, 50 μm; and margin ramp angle, 60° [21,22].

### 2.3. Grinding and Grouping

A total of 32 crowns were ground from Zirconia-reinforced Lithium Silicate (ZLS) blocks (Celtra Duo, Dentsply Sirona) using a 4-axis grinding machine (CEREC MC XL; Dentsply Sirona). A new set of burs (Step Bur 12S and Cylinder Pointed Bur 12S) was installed at the beginning of the experiment.

To analyze the effect of bur wear, the 32 crowns were divided into 4 equal groups (n = 8) based on the grinding sequence:

• **Group 1:** Crowns 1–8 (initial grinding cycle). (After milling crown 8: Step Bur 12S—73%; Cylinder Pointed Bur 12S—73%.)
• **Group 2:** Crowns 9–16. (After milling crown 16: Step Bur 12S—57%; Cylinder Pointed Bur 12S—57%.)
• **Group 3:** Crowns 17–24. (After milling crown 24: Step Bur 12S—30%; Cylinder Pointed Bur 12S—30%.)
• **Group 4:** Crowns 25–32 (final grinding cycle). (After milling crown 32: Step Bur 12S—3%; Cylinder Pointed Bur 12S—3%.)

### 2.4. Marginal Gap Measurement

**Figure 2:**
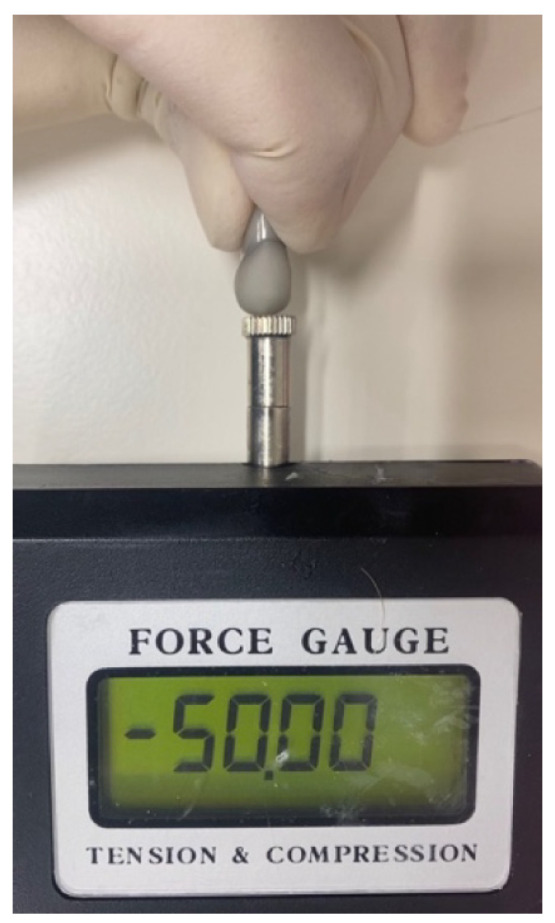
ZLS crown during cementation using Lutron.

**Figure 3:**
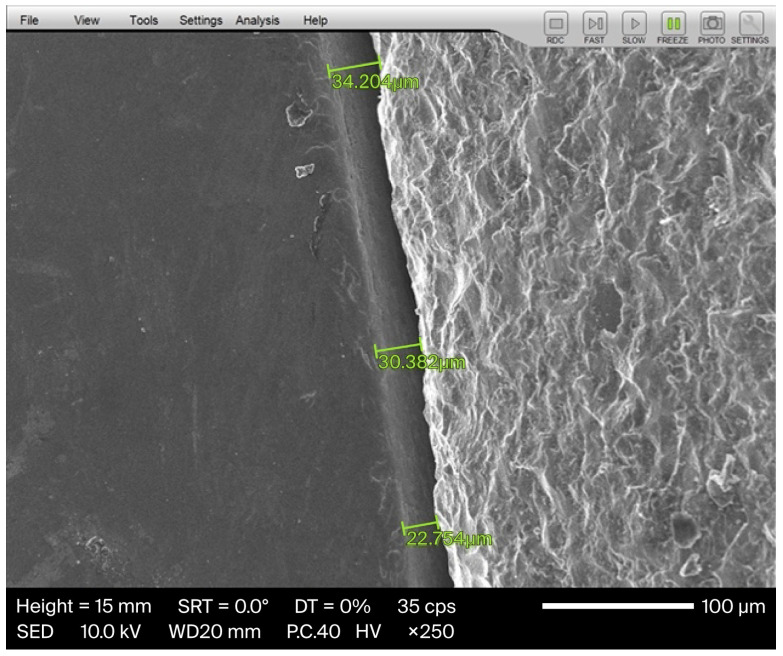
Three measurements taken under SEM at a point of interest. Green lines indicate the marginal gap at three points of measurement.

**Figure 4:**
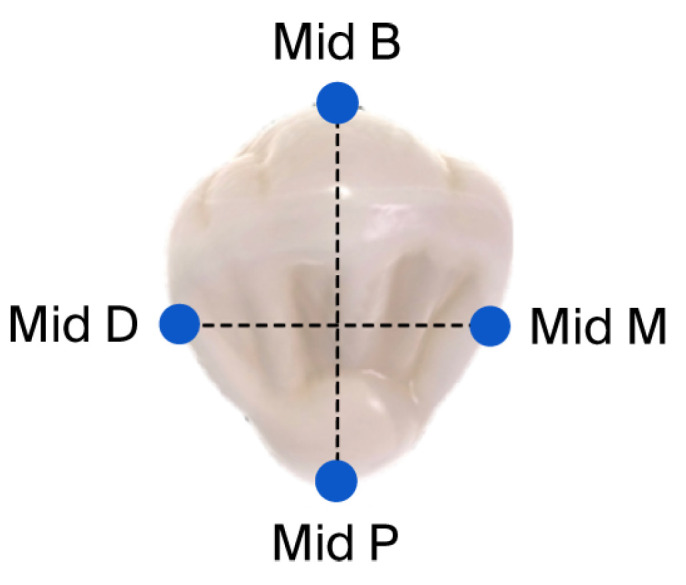
Tooth is divided into four points of interest.

Following grinding, each crown was cemented to the typodont abutment using non-eugenol temporary cement (Temp-BondTM NETM Unidose; KaVo Kerr, Brea, CA, USA) [23] (Figure 2) following the manufacturer’s protocols, and for achieving an optimal fit, a constant axial static load of 50 N was applied by a Lutron FG-20KG (Lutron Electronic Enterprise, Taipei City, Taiwan) (Figure 3) until the setting time was completed as required by the manufacturer [24]. The marginal gap was evaluated using a scanning electron microscope (SEM) (JSM-IT100; JEOL, Akishima, Tokyo, Japan) at ×250 magnification [25]. Measurements were taken at four strategic locations for each crown: mid-buccal, mid-palatal, mid-mesial, and mid-distal. At each location, 3 measurements were recorded, resulting in 12 measurements per crown [26]. Following each measurement, the crown was carefully removed manually by using a crown removal instrument (GC PLIERS, GC, Lucerne, Switzerland), and all residual temporary cement was cleaned from the typodont by using 96% ethanol. The measurement protocol was then applied for the other 31 specimens (Figure 4).

### 2.5. Statistical Analysis

The data distribution was evaluated using the Kolmogorov–Smirnov test, and consequently, a one-way analysis of variance (ANOVA) was performed to compare the effect of bur wear on the marginal gap across the four groups. The statistical significance level was set at α = 0.05. A G*power analysis indicated that a sample size of 8 crowns per group would be sensitive to the effect size of Cohen’s d = 0.626 with 80% power.

## 3. Results

The Kolmogorov–Smirnov test confirmed the normal distribution of the study variables (*p* > 0.05), and one-way ANOVA revealed no significant differences regarding the marginal gap between the surfaces: distal (*p* = 0.277), mesial (*p* = 0.732), palatal (*p* = 0.251), and buccal (*p* = 0.084). Regarding the total mean marginal gap (TMMG), no significant differences were found between the four groups (F(3,28) = 2.143, *p* = 0.117).

**Figure 5:**
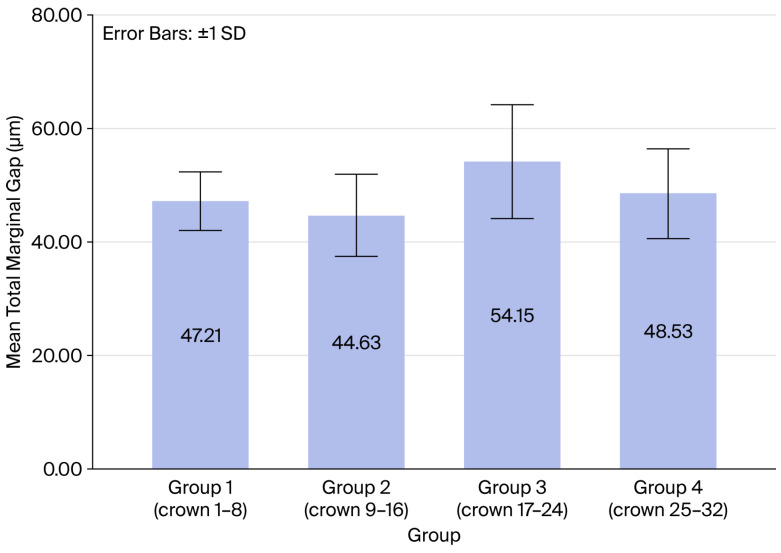
Total mean marginal gap of each group.

**Figure 6:**
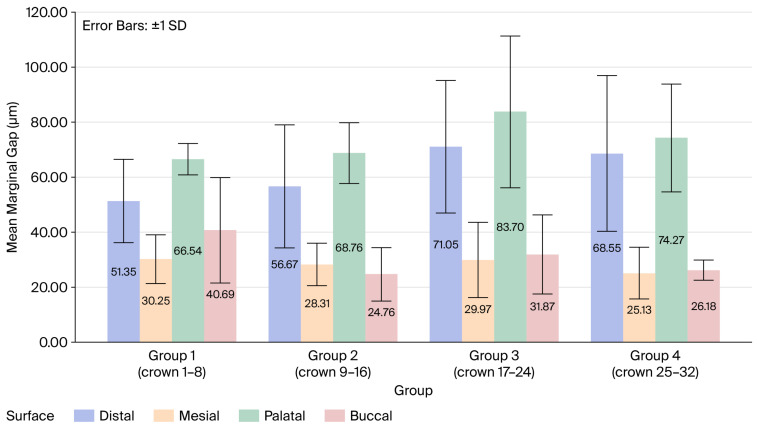
MMG of each surface for all four groups.

Group 2 exhibited the lowest mean marginal gap at 44.62 μm, while Group 3 exhibited the highest at 54.14 μm (Table 1, Figure 5), but these variations were not statistically significant. In addition, no significant differences were found regarding the mean marginal gap (MMG) between the four groups and each surface (Table 2, Figure 6).

## 4. Discussion

The purpose of this study was to assess the impact of diamond bur wear on the marginal gap of ZLS crowns fabricated using a chairside CAD/CAM system. Based on the results, the null hypothesis was accepted, as no significant differences were observed in the marginal gaps among the four groups representing progressive bur wear. Although distal and palatal surfaces exhibited higher marginal gaps than the mesial and buccal surfaces, they did not differ statistically significantly. Non-axial or eccentric loading may cause surface discrepancies, resulting in uneven marginal gaps around the crown circumference.

The marginal fit of a restoration is a key determinant of clinical success. McLean and von Fraunhofer proposed that a marginal discrepancy of up to 120 μm is clinically acceptable [18,27]. In the present study, the mean marginal gap values for all groups range between 44.62 μm and 54.14 μm, which is significantly lower than the 120 μm threshold. This indicates that even after grinding 32 units, the dimensional accuracy of the system remains high and clinically acceptable.

Interestingly, the results did not show a linear deterioration in fit with increased usage. Group 3 displayed the highest mean gap (54.14 μm), whereas Group 4, which utilized the burs at their most worn state, showed a slightly better fit (48.53 μm). This lack of a linear pattern suggests that other factors, such as inherent material variability or minor calibration shifts, might play a role, or simply that the wear level after 32 crowns is insufficient in degrading performance significantly.

In contrast to the results of this study, Tan and Dudley reported linear deterioration in the marginal gaps of LDS crowns when using both 4- and 5-axis milling machines. In their study, 12 crowns were milled after scanning with the Trios 3 scanner (3Shape, Copenhagen, Denmark), secured with removable adhesive and examined under a stereomicroscope. Different types of material (LDS), scanner (Trios 3), cement (removable adhesive), and microscope (stereo) may explain the differences in the results between the two studies [19].

Previous studies investigating the effect of CAD/CAM milling bur wear have consistently demonstrated a negative influence on restoration accuracy, primarily reflected in reduced trueness and precision. For example, Avrahami et al. reported that progressive bur wear significantly affected the accuracy of Zirconia-reinforced Lithium Silicate (ZLS) crowns when assessing 3D superimposition, although the deviations remained within clinically acceptable limits [17]. Similarly, a systematic review concluded that bur degradation adversely impacts surface quality and dimensional accuracy, with the magnitude of the effect depending on the material properties and milling system used [28]. However, most of these studies evaluated global accuracy parameters rather than marginal adaptation specifically. By contrast, investigations focusing on marginal fit across different CAD/CAM materials, including zirconia and lithium disilicate, have reported marginal discrepancies generally ranging between 40 and 120 μm, influenced by factors such as preparation design, cement spacer settings, and manufacturing systems [15,29,30].

The discrepancies between studies may be attributed to several methodological and material-related factors. First, the use of different measurement techniques such as 3D superimposition, silicone replica methods, micro-CT, or SEM introduces variability in sensitivity, with SEM being more capable of detecting small marginal discrepancies [15,30]. Second, material-specific properties play a critical role; ZLS exhibits distinct machinability and brittleness compared to zirconia, potentially making marginal areas more sensitive to tool wear [17,31]. Third, differences in CAD/CAM systems, including milling axis configuration, bur geometry, and software compensation algorithms, may either amplify or mitigate the effect of bur wear on marginal adaptation [30].

According to Dentsply Sirona, the grinding process utilizes an automated electronic bur measurement (EBM) algorithm to compensate for instrument wear. Prior to each grinding cycle, the system executes a touch-trigger probing sequence where the grinding tools (Step Bur and Cylinder Pointed Bur) interact with a designated calibration pin or the material block to determine their current axial length and radial diameter [32].

The use of ZLS (Celtra Duo) combined with the Primescan IOS and CEREC MC XL grinding unit provided precise results. The values obtained are comparable to those found in the recent literature regarding lithium disilicate crowns measured via SEM, which reported gaps in the range of 42–43 μm [29,33,34].

There are limitations to the current study that must be acknowledged. First, it was an in vitro investigation, which cannot fully simulate the complex conditions of the oral environment. Second, only one type of plastic typodont abutment, one type of restorative material (ZLS), and one type of IOS were used (Primescan), and one grinding system was evaluated. Finally, bur wear data was provided only by the machine, and visible evaluation of the burs was not performed. Future research could expand the sample size to determine the absolute failure point of the burs and investigate different ceramic materials [25,35,36,37,38].

## 5. Conclusions

Within the limitations of this study, the following conclusions can be drawn:

1. The consecutive grinding of 32 Zirconia-reinforced Lithium Silicate crowns using a single set of diamond burs did not result in a statistically significant difference in marginal adaptation.
2. The marginal gap values for all 32 crowns were well within the clinically acceptable limit of 120 μm.
3. Clinicians can confidently use a standard set of diamond burs to fabricate at least 32 ZLS single crowns without compromising the marginal integrity of the restorations when using a 4-axis milling machine.

## Figures and Tables

**Table 1:** Mean ± SD, minimum, and maximum of the total marginal gap (μm) for the four groups.

Group	N	Mean (μm)	SD (±)	Minimum	Maximum	Range
Group 1 (Crowns 1–8)	8	47.20	5.16	41.36	55.55	14.19
Group 2 (Crowns 9–16)	8	44.62	7.23	35.11	56.05	20.94
Group 3 (Crowns 17–24)	8	54.14	10.02	43.72	69.87	26.15
Group 4 (Crowns 25–32)	8	48.53	7.87	37.62	59.57	21.95

**Table 2:** Mean ± SD of marginal gap (μm) by surface.

Surface	Group 1	Group 2	Group 3	Group 4
Buccal	40.68 ± 19.17	24.76 ± 9.63	31.87 ± 14.37	26.18 ± 3.67
Palatal	66.53 ± 5.68	68.75 ± 11.02	83.70 ± 27.55	74.26 ± 19.56
Mesial	30.24 ± 8.83	28.30 ± 7.70	29.97 ± 13.63	25.13 ± 9.31
Distal	51.34 ± 15.09	56.67 ± 22.34	71.05 ± 24.02	68.54 ± 28.23

## Data Availability

The original contributions presented in this study are included in the article. Further inquiries can be directed to the corresponding author.

## Abbreviations

ZLS	zirconia-reinforced lithium silicate.
STL	standard tessellation language.
3D	3-dimensional.
CAD	computer-aided design.
CAM	computer-aided manufacturing.
IOS	intra-oral scanner.
TMMG	total mean marginal gap.
MMG	mean marginal gap.
EBM	electronic bur measurement.
